# Supplementary material for: Process evaluation of a data-driven quality improvement program within a cluster randomised controlled trial to improve coronary heart disease management in Australian primary care
Source: PLoS One. 2024 Jun 4;19(6):e0298777. doi: 10.1371/journal.pone.0298777 (PMC11149853; doi:10.1371/journal.pone.0298777)
Supplement: S1 Table — (DOCX) [file pone.0298777.s002.docx]

| **S2 Table: The 12 CHD measures for QUEL study** |
| --- |
| 1. The number of clients that are coded with a diagnosis matching the CHD definition 2. The proportion of clients with CHD where low density lipoprotein (LDL) has been measured within the previous 12 months 3. The proportion of clients with CHD whose most recent LDL result was less than 2.0 mmol/L 4. Proportion of clients with CHD with a recorded blood pressure (BP) reading taken within the previous 12 months 5. Proportion of clients with CHD whose most recent BP reading, taken within the previous 12 months, was less than or equal to 130/80 mmHg 6. Proportion of clients with CHD whose smoking status has been recorded 7. Proportion of clients with CHD recorded as a current smoker 8. Proportion of clients with CHD who are currently prescribed an anti-platelet agent 9. Proportion of clients with CHD who are currently prescribed a statin 10. Proportion of patients with CHD who are currently prescribed an ACE inhibitor or ARB 11. The proportion of clients with CHD with MBS Items 721 OR 732 claimed 12. Proportion of clients with CHD who have an influenza vaccination recorded within the previous 12 months |
