# Supplementary material for: Process evaluation of a data-driven quality improvement program within a cluster randomised controlled trial to improve coronary heart disease management in Australian primary care
Source: PLoS One. 2024 Jun 4;19(6):e0298777. doi: 10.1371/journal.pone.0298777 (PMC11149853; doi:10.1371/journal.pone.0298777)
Supplement: S2 Table — (DOCX) [file pone.0298777.s003.docx]

| **S3 Table: Monthly data extraction submitted and feedback report received.** | | | |
| --- | --- | --- | --- |
|  | **No (%) of practices submitted data (N=26)** | **No (%) practices received monthly feedback report (N=26)** | **Reasons for the inability to collect data and provide monthly feedback report** |
| **Dec-19** | 25 (96) | 25 (96) | One practice enrolled in the study in January 2020 and started submitting data from February 2020 |
| **Jan-20** | 25 (96) | 25 (96) | One practice enrolled in the study in January 2020 and started submitting data from February 2020 |
| **Feb-20** | 23 (88) | 23 (88) | Technical issues with PENCS data collection which was solved after the data submission due date for the month; therefore study team was unable to provide monthly reports to the practices |
| **Mar-20** | 26 (100) | 26 (100) | NA |
| **Apr-20** | 26 (100) | 26 (100) | NA |
| **May-20** | 26 (100) | 26 (100) | NA |
| **Jun-20** | 26 (100) | 26 (100) | NA |
| **Jul-20** | 25 (96) | 25 (96) | Reason for no data collection was not reported |
| **Aug-20** | 26 (100) | 26 (100) | NA |
| **Sep-20** | 26 (100) | 26 (100) | NA |
| **Oct-20** | 26 (100) | 26 (100) | NA |
| **Nov-20** | 26 (100) | 25 (96) | Reason for monthly feedback report not provided was not reported |
