# Supplementary material for: Process evaluation of a data-driven quality improvement program within a cluster randomised controlled trial to improve coronary heart disease management in Australian primary care
Source: PLoS One. 2024 Jun 4;19(6):e0298777. doi: 10.1371/journal.pone.0298777 (PMC11149853; doi:10.1371/journal.pone.0298777)
Supplement: S3 Table — (DOCX) [file pone.0298777.s004.docx]

| **S4 Table: Quotes summarising Quality improvement activities performed by practices** | | |
| --- | --- | --- |
| **QI activities performed by practices** | **No of practices** | **%** |
| **Use of PenCS reports to identify areas for improvement, ensured risk factors are on target by recalling patients for regular check-ups and care plans** | **18** | **69%** |
| “*We identified the CHD patients who require a CDMP and basically flag it to the GP for regular care planning*” (Practice X, Female, PM)  *“Printed reports through PenCs on BP, LDL & Smoking not recorded for Doctors to review & obtain this information in our system”* (Practice C, Female, PM)  “*Throughout this study we have found a lot of the errors in our PenCat reports came from things like coding & reasons for medication”* (Practice H, Female, PM) | | |
| **Adding reminders in patient files for GPs and other practice staff to collect and update risk factors and any other missing information including personal details** | **17** | **65%** |
| *“I would be marking the patient with QUEL so that everyone would know that this was a patient, we had to catch up on the data for and possibly do you do a care plan for just to manage them better” (Practice J, Female, PM)*  *“Addition of update information sheet for all patients at front desk to have up to date data regarding smoking status and alcohol as well as demographic info such as address and phone number”* (Practice B, Female, Nurse) | | |
| **Identifying CHD and CVD patients within the practices and creating a CHD/CVD register** | **13** | **50%** |
| **Regular data cleansing, auditing of inactive patients and recording of data correctly to improve data quality** | **13** | **50%** |
| “*Inactivating patients on the register who live overseas or interstate or who have other regular GPs or those presenting for immunisation only*” (Practice F, Female, GP) *“We chose to use Top Bar as an assistance tool to ensure the missing information was recorded properly, this is done through Top Bar Prompts”* (Practice H, Female, PM) “*One of the things I did regularly was with all patients who had free text or incorrect diagnosis recorded, I would ask the doctors to change or I would change it to make sure that it was getting captured*” (Practice J, Female, PM) | | |
| **Team Activity – regular team meetings, allocating responsibilities to all, getting experienced staff on board.** | **13** | **50%** |
| *“We posted results on blackboards in tea room the progressive target results to encourage team to implement QI*” (Practice D, Female, PM)  *“Hired a Nurse for 2 days a week to review CVD & CHD patients, check eligibility for Care Plans & Assessments, recalled patients in for appointment with Nurse to update data and sent to doctors for pathology after seeing Nurse”* (Practice C, Female, Nurse)  *“We are all working together at the practice as a team, everyone has made a concerted effort to improve our data quality and we are particularly working on increasing our care plans for patients that meet the cohort criteria”* (Practice Q, Female, PM) | | |
| **Educate patients on heart health, smoking cessation, lifestyle modification and self-management of CHD at home** | **10** | **38%** |
| *“We have sheets set up within our database, where we would have targets for patients particularly those with that already had non-destructive cardiovascular disease. We gave those patients a handout to take home and say look, with the risk factors you have got, this is where we'd like your target to be”* (Practice V, Female, Nurse) | | |
| **Implemented new processes to improve care of CHD patients** | **7** | **27%** |
| *“Developed a template to collect height, weight, family history, smoking, alcohol for all patients upon arrival of patients”* (Practice D, Female, PM)  *“We scanned 20,000 patient paper files into the computer so we are paperless*” (Practice C, Female, PM)  *“I lead the creation of a Cardiovascular Disease Prevention clinic called the 'Healthy Heart clinic' to regularly check CHD patients with the practice nurse and practice manager”* (Practice W, Female, GP registrar) | | |
| **Review of current processes within the practices to reflect CHD QI changes** | **7** | **27%** |
| *“We created a letter for patients that encourages participation rather than another bland invite/recall letter and sent letters to eligible patients over 3 months”* (Practice B, Female, Nurse) *“Constantly reviewing progress against our targets, changing process to achieve targets dependent upon the progress”* (HC, Female, GP) | | |
| **Identified CHD patients who did not have care plans, required review of care plans, had a recent event thus ensured more eligible patients received care plans** | **6** | **23%** |
| *“So my care coordinator was tasked to ensure that whenever she sees a patient with CHD, flag it to the GP to organise a care plan if eligible”* (Practice X, Female, PM) | | |
| *CHD: Coronary heart disease, CDMP: Chronic disease management plan, GP: General practitioner, PM: Practice manager, BP: Blood pressure, LDL: Low-density lipoprotein, CVD: Cardiovascular disease. | | |
