# Supplementary material for: Process evaluation of a data-driven quality improvement program within a cluster randomised controlled trial to improve coronary heart disease management in Australian primary care
Source: PLoS One. 2024 Jun 4;19(6):e0298777. doi: 10.1371/journal.pone.0298777 (PMC11149853; doi:10.1371/journal.pone.0298777)
Supplement: S2 Checklist — (DOCX) [file pone.0298777.s006.docx]

STROBE Statement—checklist of items that should be included in reports of observational studies

|  | Item No. | Recommendation | Page  No. | Relevant text from manuscript |
| --- | --- | --- | --- | --- |
| **Title and abstract** | 1 | (*a*) Indicate the study’s design with a commonly used term in the title or the abstract | 3 | The study aimed to evaluate primary care practices’ engagement with a quality improvement intervention for patients with coronary heart disease in Australia. |
|  |  | (*b*) Provide in the abstract an informative and balanced summary of what was done and what was found | 3 | The study found that practice engagement in the intervention was common, with learning workshops and monthly feedback reports identified as the most useful features.  Introduction |
| Introduction | | | |  |
| Background/rationale | 2 | Explain the scientific background and rationale for the investigation being reported | 4-5 | Primary care plays a crucial role in reducing the burden of coronary heart disease (CHD). The Australian government introduced the Practice Incentive Program Quality Improvement (PIP-QI) to enhance the management and quality of care for people with chronic diseases, including CHD. However, more research is needed to understand how practices can best implement PIP-QI to improve care in CHD. |
| Objectives | 3 | State specific objectives, including any prespecified hypotheses | 5 | (i) describe and analyse practice engagement, attendance, time commitment, skills and capacity of the practice team members associated with the intervention; and (ii) explore to what extent the intervention was delivered as intended, whether the intervention features were useful. |
| Methods | | | |  |
| Study design | 4 | Present key elements of study design early in the paper | 6 | For this study, a process evaluation was performed on the intervention practices using a mixed-methods approach, collecting both quantitative and qualitative data from 27 primary care practices. |
| Setting | 5 | Describe the setting, locations, and relevant dates, including periods of recruitment, exposure, follow-up, and data collection | 6 and 8 | The study was conducted in 27 urban and rural primary care practices of varying sizes within 10 PHNs and across four Australian states (New South Wales, South Australia, Victoria and Queensland).  The QUEL intervention was delivered between November 2019 and November 2020  Data collection described on page 8 |
| Participants | 6 | (*a*) *Cohort study*—Give the eligibility criteria, and the sources and methods of selection of participants. Describe methods of follow-up  *Case-control study*—Give the eligibility criteria, and the sources and methods of case ascertainment and control selection. Give the rationale for the choice of cases and controls  *Cross-sectional study*—Give the eligibility criteria, and the sources and methods of selection of participants | 6 | It is a mixed-methods study. Therefore, Participants were included if they were (i) team members from a practice randomised to receive the intervention including general practitioners (GPs), nurses and practice managers (PM), (ii) PHN staff who provided direct support to intervention practices, and (iii) provided written informed consent. |
|  |  | (*b*) *Cohort study*—For matched studies, give matching criteria and number of exposed and unexposed  *Case-control study*—For matched studies, give matching criteria and the number of controls per case |  | NA |
| Variables | 7 | Clearly define all outcomes, exposures, predictors, potential confounders, and effect modifiers. Give diagnostic criteria, if applicable | 9 | The outcomes were practice engagement with the QI intervention. Which was defined as attendance in workshops, PDSA submission and use of SharePoint. Other variables included skills and capacity of practice team members, and time commitment. |
| Data sources/ measurement | 8* | For each variable of interest, give sources of data and details of methods of assessment (measurement). Describe comparability of assessment methods if there is more than one group | 8 | Data from the following sources were synthesised to evaluate the QI intervention and address the study aims 1) practice-level enrolment data, 2) attendance record, 3) SharePoint resources, 4) practice correspondence record, 5) data collection record, 6) PDSA cycles, 7) learning workshop surveys, 8) end-of-program evaluation survey and 9) semi-structured interviews of practice team members and PHN Staff. |
| Bias | 9 | Describe any efforts to address potential sources of bias | 6 and 8 | The study aimed to address potential sources of bias through rigorous data collection. |
| Study size | 10 | Explain how the study size was arrived at | 6 | The cluster randomised controlled trial included 52 practices. The practices were then randomised into intervention and control group. The intervention was delivered to 27 of the 52 practices. Therefore, the process evaluation included a study size of 27 intervention practices. |

Continued on next page

| Quantitative variables | 11 | Explain how quantitative variables were handled in the analyses. If applicable, describe which groupings were chosen and why | 9-10 | Descriptive statistics were used to analyse quantitative data. Responses and measurements from all data sources are presented as numbers and percentages for categorical variables and mean and standard deviation (SD) or median and interquartile intervals (IQI) for continuous variables. |
| --- | --- | --- | --- | --- |
| Statistical methods | 12 | (*a*) Describe all statistical methods, including those used to control for confounding | 9-10 | Descriptive statistics were used to analyse quantitative data. Responses and measurements from all data sources are presented as numbers and percentages for categorical variables and mean and standard deviation (SD) or median and interquartile intervals (IQI) for continuous variables.  Qualitative data from semi-structured interviews, surveys and other data sources were analysed using thematic analysis |
|  |  | (*b*) Describe any methods used to examine subgroups and interactions |  | N/A |
|  |  | (*c*) Explain how missing data were addressed | 10 | Practices which didn’t respond to the survey were not included in the analysis |
|  |  | (*d*) *Cohort study*—If applicable, explain how loss to follow-up was addressed  *Case-control study*—If applicable, explain how matching of cases and controls was addressed  *Cross-sectional study*—If applicable, describe analytical methods taking account of sampling strategy |  | N/A |
|  |  | (*e*) Describe any sensitivity analyses |  | N/A |
| Results | | | | |
| Participants | 13* | (a) Report numbers of individuals at each stage of study—eg numbers potentially eligible, examined for eligibility, confirmed eligible, included in the study, completing follow-up, and analysed | 10-11 | Participants results presented in page 10 also summarised in Table 1: Summary of participants providing feedback on learning workshop surveys |
|  |  | (b) Give reasons for non-participation at each stage | 9 | One practice withdrew following participation in the first learning workshop due to staff change and was excluded from the analysis. |
|  |  | (c) Consider use of a flow diagram |  | N/A |
| Descriptive data | 14* | (a) Give characteristics of study participants (eg demographic, clinical, social) and information on exposures and potential confounders | 14-15 | Characteristics of practice team members are described in Table 3: Characteristics of practice team members leading the QI activities in the intervention practices |
|  |  | (b) Indicate number of participants with missing data for each variable of interest | 12-14 | Indicated by the denominator, in Table 2 and also described in paragraph titled ‘Use of SharePoint’ on page 14. |
|  |  | (c) *Cohort study*—Summarise follow-up time (eg, average and total amount) |  | NA |
| Outcome data | 15* | *Cohort study*—Report numbers of outcome events or summary measures over time | 9 | Outcome measures reported |
|  |  | *Case-control study—*Report numbers in each exposure category, or summary measures of exposure |  |  |
|  |  | *Cross-sectional study—*Report numbers of outcome events or summary measures |  |  |
| Main results | 16 | (*a*) Give unadjusted estimates and, if applicable, confounder-adjusted estimates and their precision (eg, 95% confidence interval). Make clear which confounders were adjusted for and why they were included | 10-23 | Main results of the study described under Results section |
|  |  | (*b*) Report category boundaries when continuous variables were categorized |  | NA |
|  |  | (*c*) If relevant, consider translating estimates of relative risk into absolute risk for a meaningful time period |  | NA |

Continued on next page

| Other analyses | 17 | Report other analyses done—eg analyses of subgroups and interactions, and sensitivity analyses |  | NA |
| --- | --- | --- | --- | --- |
| Discussion | | | | |
| Key results | 18 | Summarise key results with reference to study objectives | 23-24 | Although most practices reported their quality of care for CHD patients has improved because of their participation in the QI intervention, practice engagement was varied. Attendance at learning workshops ranged between 50–77%, engagement with the PDSA cycle was low, with only 12% of practices submitting ten cycles or more over 12 months. Qualitative analysis identified team members in a leading role or with clinical backgrounds were able to implement QI changes more effectively within their practices and practices regularly set aside additional time during working hours to implement these changes. The study also identified learning workshops and monthly feedback reports as the two key useful intervention features to facilitate QI changes. |
| Limitations | 19 | Discuss limitations of the study, taking into account sources of potential bias or imprecision. Discuss both direction and magnitude of any potential bias | 26 | Study strengths and limitations described in page 26 on the manuscript |
| Interpretation | 20 | Give a cautious overall interpretation of results considering objectives, limitations, multiplicity of analyses, results from similar studies, and other relevant evidence | 23-26 | Described in pages 23 – 26 |
| Generalisability | 21 | Discuss the generalisability (external validity) of the study results | 25-26 | The practices included in the evaluation were from various sizes and regions ensured wider representation of participants, therefore enhancing generalisability of the findings to similar healthcare setting. The intervention features and implementation strategies described in this study can be used as a useful framework to be replicated in similar healthcare settings aiming to enhance the quality of care for patients with chronic diseases. |
| Other information | |  | | |
| Funding | 22 | Give the source of funding and the role of the funders for the present study and, if applicable, for the original study on which the present article is based | 29 | Funding information provided on an individual paragraph titles ‘Funding disclosure’ on page - 29 |

*Give information separately for cases and controls in case-control studies and, if applicable, for exposed and unexposed groups in cohort and cross-sectional studies.

**Note:** An Explanation and Elaboration article discusses each checklist item and gives methodological background and published examples of transparent reporting. The STROBE checklist is best used in conjunction with this article (freely available on the Web sites of PLoS Medicine at http://www.plosmedicine.org/, Annals of Internal Medicine at http://www.annals.org/, and Epidemiology at http://www.epidem.com/). Information on the STROBE Initiative is available at www.strobe-statement.org.
