# Supplementary material for: Process evaluation of a data-driven quality improvement program within a cluster randomised controlled trial to improve coronary heart disease management in Australian primary care
Source: PLoS One. 2024 Jun 4;19(6):e0298777. doi: 10.1371/journal.pone.0298777 (PMC11149853; doi:10.1371/journal.pone.0298777)
Supplement: Attachment — Submitted filename: Response to reviewers.docx [file pone.0298777.s007.docx]

When submitting your revision, we need you to address these additional requirements.

1. Please ensure that your manuscript meets PLOS ONE's style requirements, including those for file naming. The PLOS ONE style templates can be found at
   <https://journals.plos.org/plosone/s/file?id=wjVg/PLOSOne_formatting_sample_main_body.pdf> and <https://journals.plos.org/plosone/s/file?id=ba62/PLOSOne_formatting_sample_title_authors_affiliations.pdf>

Response: Formatting checked throughout the manuscript.

2. We note that the grant information you provided in the ‘Funding Information’ and ‘Financial Disclosure’ sections do not match. When you resubmit, please ensure that you provide the correct grant numbers for the awards you received for your study in the ‘Funding Information’ section.

Response: The financial information “Funding for this study was provided by a National Health and Medical Research Council (NHMRC) Partnership Project Grant (Award Grant Number: GNT1140807). Additional in-kind and cash support from the following partner organisations; Amgen (cash support), Austin Health, Australian Cardiovascular Health and Rehabilitation Association, Australian Commission on Safety and Quality in Health Care, Australian Primary Health Care Nurses Association, Brisbane South PHN, Fairfield General Practice Unit, Heart Support Australia, Improvement Foundation, Inala Primary Care, National Heart Foundation of Australia, Nepean Blue Mountains PHN (cash support), Royal Australian College of General Practitioners, Sanofi (provided cash support via the Externally Sponsored Collaboration pathway), South Western Sydney PHN, The George Institute for Global Health (cash support) and University of Melbourne” has been included in the cover letter to be updated.

3. Thank you for stating the following financial disclosure: “Funding for this study was provided by a National Health and Medical Research Council (NHMRC) Partnership Project Grant (Award Grant Number: GNT1140807). Additional in-kind and cash support from the following partner organisations; Amgen (cash support), Austin Health, Australian Cardiovascular Health and Rehabilitation Association, Australian Commission on Safety and Quality in Health Care, Australian Primary Health Care Nurses Association, Brisbane South PHN, Fairfield General Practice Unit, Heart Support Australia, Improvement Foundation, Inala Primary Care, National Heart Foundation of Australia, Nepean Blue Mountains PHN (cash support), Royal Australian College of General Practitioners, Sanofi (provided cash support via the Externally Sponsored Collaboration pathway), South Western Sydney PHN, The George Institute for Global Health (cash support) and University of Melbourne. JR is funded by a NHMRC Investigator Grant (GNT1143538). KH is supported by the NHMRC Investigator Grant (Emerging leadership 1) (APP1196724). MW is supported by the NHMRC grants (1080206 and 1149987). CR is supported by a NHMRC Principal Research Fellowship (APP1136372). TL is funded by a NHMRC Early Career Fellowship (APP110230). EA is supported by a National Heart Foundation Australia postdoctoral fellowship (101884). CC’s salary is funded by a Career Development Fellowship level 2 co-funded by the NHMRC and National Heart Foundation Future Leader Award (APP1105447), which supports 0.05FTE for trial meetings."

Please state what role the funders took in the study.  If the funders had no role, please state: ""The funders had no role in study design, data collection and analysis, decision to publish, or preparation of the manuscript.""

If this statement is not correct you must amend it as needed. 
Please include this amended Role of Funder statement in your cover letter; we will change the online submission form on your behalf.

Response – The following statement, “Role of Funder - The funders, including funding body and industry partners, had no role in study design, data collection and analysis, decision to publish, or preparation of the manuscript.” has been included in the cover letter to be updated.

4. Thank you for stating the following in the Competing Interests section:
   "The funding body and industry partners was not involved in the design of the study; and will not have any role during its execution, analyses, interpretation of the data, or decision to submit results. Amgen and Sanofi Australia has provided cash support to the main study. MW is a consultant to Amgen, Freeline and Kyowa Kirin. Other authors havenothing to disclose."

Please confirm that this does not alter your adherence to all PLOS ONE policies on sharing data and materials, by including the following statement: ""This does not alter our adherence to PLOS ONE policies on sharing data and materials.” (as detailed online in our guide for authors <http://journals.plos.org/plosone/s/competing-interests>).  If there are restrictions on sharing of data and/or materials, please state these. Please note that we cannot proceed with consideration of your article until this information has been declared.

Please include your updated Competing Interests statement in your cover letter; we will change the online submission form on your behalf.

Response – The following “Competing Interest Statement - The funding body and industry partners were not involved in the design of the study and did not have any role during its execution, analyses, interpretation of the data, or decision to submit results. Amgen and Sanofi Australia have provided cash support to the main study. MW is a consultant to Amgen, Freeline and Kyowa Kirin. Other authors have nothing to disclose. This does not alter our adherence to PLOS ONE policy on sharing data and materials” has been included in the cover letter to be updated.

5. We note that you have indicated that data from this study are available upon request. PLOS only allows data to be available upon request if there are legal or ethical restrictions on sharing data publicly. For more information on unacceptable data access restrictions, please see http://journals.plos.org/plosone/s/data-availability#loc-unacceptable-data-access-restrictions.

In your revised cover letter, please address the following prompts:

a) If there are ethical or legal restrictions on sharing a de-identified data set, please explain them in detail (e.g., data contain potentially sensitive information, data are owned by a third-party organization, etc.) and who has imposed them (e.g., an ethics committee). Please also provide contact information for a data access committee, ethics committee, or other institutional body to which data requests may be sent.

Response – The following “Data availability statement: The study data cannot be publicly shared due to the ethical restrictions on sharing potentially identifiable information, which may compromise the privacy of the participants, according to the NSW Population and Health Services Research Ethics Committee (CINSW). The participant consent form does not include public deposition of the data. Data is stored securely in the University of Sydney’s Research Data Storage Database. However, in accordance with PLoS One guidelines, de-identified data can be made available upon request from the ethics committee (NSW Population & Health Services Research Ethics Committee). Contact information to request data: [cinsw-ethics@health.nsw.gov.au](mailto:cinsw-ethics@health.nsw.gov.au).” has been included in the cover letter to be updated.

b) If there are no restrictions, please upload the minimal anonymized data set necessary to replicate your study findings as either Supporting Information files or to a stable, public repository and provide us with the relevant URLs, DOIs, or accession numbers. For a list of acceptable repositories, please see <http://journals.plos.org/plosone/s/data-availability#loc-recommended-repositories>.

We will update your Data Availability statement on your behalf to reflect the information you provide.

6. Your ethics statement should only appear in the Methods section of your manuscript. If your ethics statement is written in any section besides the Methods, please delete it from any other section.

Response – Ethics statement from page 35 has been deleted.

Additional Editor Comments:

Please carefully address the issues raised in the comments and, up front in your revised paper. Your revised paper will be sent to the same reviewers, as well as possibly new reviewers, for evaluation.

7. Make sure the Abstract briefly describes the paper as it is used in abstracting and citation services. Clearly specify the Purpose, Methodology, and problem findings.

Response - Abstract has been updated throughout. Please see pages 3-4, lines 55 – 81 of the revised manuscript.

8. Highlight the major spatial modulation objective in section 1. What is your work contribution in this area.

Response – The following sentences have been edited and added in section 1 - “The primary objective of this study is to comprehensively evaluate the QUEL intervention by examining practice engagement, providing insight into the intervention and delivery and assessing the usefulness of the intervention features from healthcare providers perspective. Specifically, it aims to (i) describe and analyse practice engagement, time commitment, skills and capacity of the practice team members associated with the intervention; and (ii) explore to what extent the intervention was delivered as intended, whether the intervention features were useful. We hypothesise that higher practice engagement and perceived usefulness of intervention features are positively associated with the adoption of data-driven QI strategies in improving the care of patients with CHD in primary care practices.” Please see page 6, lines 124 – 136 of the revised Manuscript.

9. Spell out each acronym the first time used in the body of the paper. Spell out acronyms in the Abstract only if used there.

Response – Acronyms have been spelled out the first time used in the body of the paper. Acronyms have been spelled out in the Abstract, only if used there. Please see pages 3-4, lines 55-81 of the revised manuscript.

10. You may ignore any suggestion of including self-references by reviewers if not applicable.

Response – Not applicable; no reviewers requested self-references.

11. Make sure that the Conclusion briefly summarizes the results of the paper it should not repeat phrases from the Introduction. Keep the Conclusion to about 200 words. Do not use any references or acronyms in the Conclusion.

Response – The conclusion has been updated to summarise the results of the paper. Please see pages 33 - 34, lines 630 - 647 of the revised manuscript.

12. Make sure all figures and tables are referred to in the body of the paper.

Response – All figures and tables have been referred to throughout the body of the manuscript.

13. Tabulate the major drawbacks and problem identification under section 2. Also relate the problem with your proposed approach.

Response – Challenges faced during the study and their proposed solutions are discussed in details within section 4 as limitations. Please see pages 31 - 32, lines 586 – 607 of the revised manuscript.

14. Explain the section 3 with proper diagrammatic approach.

Response – A flow diagram of the process evaluation has been developed using VISIO and has been referred as Fig 1 on page 7 of the revised manuscript. Please see attached Fig 1.

15. In section 4, Give some real time industrial case study approach to elaborate your proposed approach.

Response – Case studies have been added throughout section 4. Please see pages 28 – 30, lines 516 – 567 of the revised manuscript.

16. It is recommended to use a professional proofread and native English correction. Papers with less than excellent English will not be published even if technically perfect.

Response – Professional proofreading (Grammarly) and native English correction have been used in the manuscript.

17. Remove all unwanted and self-citations from the reference section.

Response – All unwanted and self-citations have been removed from the reference section.

[Note: HTML markup is below. Please do not edit.]

Reviewers' comments:

Reviewer's Responses to Questions

**Comments to the Author**

1. Is the manuscript technically sound, and do the data support the conclusions?

   The manuscript must describe a technically sound piece of scientific research with data that supports the conclusions. Experiments must have been conducted rigorously, with appropriate controls, replication, and sample sizes. The conclusions must be drawn appropriately based on the data presented.

Reviewer #1: Yes

Reviewer #2: Partly

2. Has the statistical analysis been performed appropriately and rigorously?

Reviewer #1: Yes

Reviewer #2: No

Have the authors made all data underlying the findings in their manuscript fully available?

The [PLOS Data policy](http://www.plosone.org/static/policies.action#sharing) requires authors to make all data underlying the findings described in their manuscript fully available without restriction, with rare exception (please refer to the Data Availability Statement in the manuscript PDF file). The data should be provided as part of the manuscript or its supporting information, or deposited to a public repository. For example, in addition to summary statistics, the data points behind means, medians and variance measures should be available. If there are restrictions on publicly sharing data—e.g. participant privacy or use of data from a third party—those must be specified.

Reviewer #1: Yes

Reviewer #2: No

Is the manuscript presented in an intelligible fashion and written in standard English?

PLOS ONE does not copyedit accepted manuscripts, so the language in submitted articles must be clear, correct, and unambiguous. Any typographical or grammatical errors should be corrected at revision, so please note any specific errors here.

Reviewer #1: Yes

Reviewer #2: Yes

3. Review Comments to the Author

Please use the space provided to explain your answers to the questions above. You may also include additional comments for the author, including concerns about dual publication, research ethics, or publication ethics. (Please upload your review as an attachment if it exceeds 20,000 characters)

Reviewer #1: The article is well written with detailed statistics of the process evaluation but I found that the major limitation was a lack of actionable new information that would be useful to clinicians or researchers. It may be helpful to emphasise this more in the discussion.

Reviewer #2: This paper aims to report the process evaluation of a data-driven quality improvement program as part of a trial (the QUEL program) to improve the management of coronary heart disease (CHD) in primary care in Australia. The paper has the potential to make a useful contribution because we need to know more about the best ways to implement interventions in the complicated world of primary healthcare. However, the paper is not clear enough about its main focus, does not report the statistics in sufficient detail, and lacks information about key aspects of the process that would have implications for the wider roll-out of the intervention beyond the trial (information which you might expect to get from a process evaluation). These things make it hard to be sure that the conclusions drawn are warranted by the findings.

but I found that the major limitation was a lack of actionable new information that would be useful to clinicians or researchers. It may be helpful to emphasise this more in the discussion.

Response: Section 4 (Discussion) has been edited to include new information. Please see pages 27 – 33, lines 489 – 627 of the revised manuscript.

Focus
It is important to separate the presumed primary outcome of the trial, which I understand to be a positive change in the management of CHD as evidenced by the 12 measures given in the S1 Table, from the focus of the process evaluation reported in this paper. An improvement in CHD management is predicted to arise from iterative improvements in these 12 measures using a data-driven quality improvement paradigm. In other words, the purpose of the trial was to encourage primary care practices to use quality improvement methods to improve their performance on these measures successively over a 12-month period. Thus a key aspect of the process evaluation would be to understand whether the practices did indeed focus on these data and make improvements. However, the outcome measures for this paper were defined as attendance at workshops, submission of PDSA cycles and use of SharePoint (p.9). Time commitment and skills of the practice team members were also recorded, but there do not appear to be any outcomes that are clearly related to the use of data to drive the quality improvement. It is possible that the submission of PDSA cycles is the proxy for this, but the paper does not describe any examples of how practices designed PDSA cycles to make improvements in the measures, and the data for workshop attendance is not linked to the data on PDSA submission (i.e., we do not know whether practices with higher attendance at workshops submit more PDSA cycles).

So, the paper needs to be clearer about which processes are being evaluated and the measures being used.

Response –

1. We have included the following sentence “We hypothesise that higher practice engagement, attendance, and time commitment of primary care practice team members and perceived usefulness of intervention features are positively associated with the adoption of data-driven QI strategies in improving CHD care in primary care practices” under the Introduction section. Please see page 6, lines 133 - 136 of the revised manuscript.
2. The following sentences “It was beyond the scope of the process evaluation to evaluate whether the intervention was effective in improving the pre-defined CHD. However, the QUEL cRCT will perform an evaluation to analyse the effectiveness of the QI intervention on the clinical outcomes of the study, including the CHD performance measures.” as a limitation. Please see page 32, lines 604 – 607 of the revised manuscript.
3. The following sentences “Table 2 reveals cross-tabulation of workshop attendance with PDSA submissions and SharePoint use revealing a pattern in practice engagement. PDSA submission and SharePoint use was balanced for practices which attended five or more workshops. In contrast, practices which attended less than two workshops revealed low use” were added on page 15, lines 342 – 346 and “Additionally, table 3 provides a detailed summary of practice engagement with the intervention over the 12- months among participating practices. Overall, 11 practices that attended five or more learning workshops submitted 57 PDSAs and the median (IQI) of SharePoint use was 231 (150.5, 338.5). Another 11 practices that attended three to four workshops submitted 27 PDSAs and the median (IQI) of SharePoint use was 139 (110, 169.5). Four practices that attended less than 3 workshops, submitted 13 PDSAs with median (IQI) of SharePoint use 168.5 (91.5, 220.2)” on page 16, lines 348 – 354 of the revised manuscript.
4. The following sentences are edited and added “Almost 70% (18/26) of the practices reported higher time commitment that they spent a considerable amount of timeon using the electronic health system within working hours to identify CHD patients, monitor and track patients, develop care plans and record keeping. Table 5 presents a cross tabulation of these practices on their engagement with the intervention. Additionally, two practices reported moderate level of time commitment to perform QI activities. Both practices attended five or more learning workshops, submitted 14 PDSAs and the median (IQI) of SharePoint use was 154. 19% (5/26) of the practices that didn’t report any data on time commitment showed varying levels of engagement. One practice attended five or more workshops, submitted one PDSA and visited the SharePoint site 50 times. Two practices attended three to four workshops, submitted 11 PDSAs and the median (IQI) of SharePoint use was 179. Three practices that attended less than three workshops submitted 7 PDSAs, and the median (IQI) of SharePoint use was 225.” Please see pages 18 - 19, lines 380 – 392.

Statistics
I found much of the presentation of descriptive statistics to be rather unsophisticated and, as suggested above, much more could be learnt by some cross-tabulation of the frequencies. So, submission of PDSA cycles and use of analytics could have been broken down by attendance at workshops, and staff category could be cross-tabulated with years of experience and gender.

In the text accompanying each table, there tends to be a lot of repetition of the data that is already reported in the table which is unnecessary and tedious to read. Cross-tabulation would allow more nuanced comments to be drawn about the relationships and differences.

Response: Tables 2, 3, and 5 are now added to the manuscript. Please see pages 15, 16 – 17 and 19 of the revised manuscript.

There is also a lack of detail and precision in reporting some data, e.g., the size of primary care practices is described as 'varying',

Response – The following sentences were added “Twenty-six primary care practices from four Australian states (69% of the practices from New South Wales, 15% from Victoria, 12% from South Australia and 4% from Queensland) participated in the QUEL intervention. Among these practices, six were located in rural and the remaining 22 were in urban areas across these four states. The practices were also of varying size, with the number of GPs varying from 1 – 18 and the median (IQI) number of GPs in these practices were 7 (6.25).” Please see page 12, lines 281 – 286 of the revised manuscript.

data gathering was conducted "using a variety of sources including learning workshop surveys, attendance records, PDSAs, among others"

Response: The data sources are all described in the published protocol, which has been included as supplementary information as S1 Appendix and referenced in the manuscript by including the following sentence: “Details of the data sources are published elsewhere (S1).^21^” Please see page 9, lines 203 – 204 of the revised manuscript.

interview participants "were selected based on factors such as gender, location, and practice performance". In each of these cases, more detail needs to be provided.

Response: The following sentences were edited: “To ensure a balanced representation and minimise bias, team members for the semi-structured interviews were invited to participate from both rural and urban practices and from practices representing high, medium or low attendance in the learning workshops.” Please see page 9, lines 211 – 215 of the revised manuscript.

In addition the use of range categories reduces the level of information. For instance, in Table 2 why not use a frequency chart to display how many practices attended 1,2,3, etc. workshops? Or similarly to display the number of practices submitting different numbers of PDSA cycles over the 12 month period?

Response – individual frequency graphs have been included for workshop attendance, PDSA submission and SharePoint use and referred as Fig 2 on page 14 of the revised manuscript. Please see attached Fig 2.

In the qualitative data, each quote should be attributed to a practice ID, as well as the gender and staff category. This is so that we know that the quotes come from a range of practices.

Response – Practice ID is included for all quotes throughout the manuscript and in S4 Table.

The extra sophistication of analysis and detail would need to be reflected in the points made in the Discussion section.

Response – the following sentences “This study elaborates on the primary care practices’ engagement with the QI intervention in improving care of CHD. The Intervention led to increased scope of QI activities for the practice team members leading to more than half of the practices (69%) self-reporting improvement in their QI activities for CHD patient care. As a result, majority (73%) of the practices felt they were ready for PIP-QI and some (42%) were able to claim the benefits. However, the practice engagement with the intervention was varied. Engagement with the PDSA cycles was low with only 12% of practices submitting 10 or more cycles over 12 months. However, the submission range of PDSA cycles was diverse (0-10) in all practices despite the varied attendance. Practices that attended a higher number of learning workshops submitted higher number of PDSA cycles and showed higher engagement with the SharePoint site. However, variations were seen within each attendance category, indicating several factors contributing to the different engagement level. Additionally, practices reporting higher time commitment generally demonstrated higher learning workshop attendance, submitted more PDSAs, and used SharePoint more, suggesting a positive correlation between time commitment and engagement in the intervention with some variations in a small portion of the practices. Qualitative analysis identified team members in a leading role or with clinical backgrounds were able to implement QI changes more effectively within their practices and practices regularly set aside additional time during working hours to implement these changes. The study also identified learning workshops and monthly feedback reports as the two key useful intervention features to facilitate QI activities and changes.” has been included. Please see pages 27 – 28, lines 489 – 514 of the revised manuscript.

Implications for wider roll-out
The trial that is the subject of this process evaluation appears to be an efficacy trial rather than an effectiveness trial and although there are several occasions when the authors describe the high level of support provided by the trial team, they also conclude "we were unable to obtain comprehensive data regarding the extent of support provided by both the PHNs and study team during the intervention period". Clearly, an important part of the process evaluation would be to understand whether a high level of support is going to be needed to introduce the intervention on a wider scale and ensure fidelity with the intervention; a level of support which may be unrealistic. However, this paper is not able to report on this, which rather weakens its contribution. At the very least, the authors might have considered the implications of this lack of information.

Response – Following sentences: “Findings from the study suggest that external factors, unexpected events or occurrences should be taken into consideration when planning broader implementation strategies. We also acknowledge that the paper could benefit from a more detailed exploration of PDSAs in understanding a direct association between workshop attendance and PDSA submissions with the intervention. Highlighting a scope for future research to explore the various factors associated with the PDSA engagement.^[44]^ Adding a more robust, sophisticated, and accurate data collection and analysis method could provide more nuanced measures of the findings, therefore enhancing the feasibility of future studies. The limitation in accurately assessing the level of support required for the successful implementation can be addressed by establishing an effective collaboration and incorporating remote reporting and data collection between the research team and the PHNs.^[36]^ Furthermore, enabling tailored support and addressing the nuanced dynamics of time commitment is important to optimise engagement with QI interventions across diverse practices. The use of these combined strategies, along with ongoing training, designating clinicians as QI champions and increased use of data-driven technology to monitor progress, can collectively contribute towards the large-scale roll-out of future QI programs with an aim to improve care for patients with CHD across diverse healthcare settings.^[45, 46]^” have been included in Section 4 to include implications of wider – rollout of the intervention. Please see pages 32 - 33, lines 609 – 627 of the revised manuscript.

Minor points
line 127 - it is not clear what is meant by receiving usual care here.

Response – The following sentence “The QUEL study is a cluster randomised trial, where primary care practices were randomised to receive the QI intervention or continued to receive usual care without access to the intervention during the study. However, control practices were offered an opportunity to participate in a series of virtual workshops after the completion of 24 months data collection” has been edited. Please see page 6, lines 141 – 145 of the revised manuscript.

lines 136-9 - are these inclusion criteria additive or alternatives?

Response: The following sentence has been edited “Participants were included if they met any of the following criteria: (i) team members from a practice randomised to receive the intervention, including general practitioners (GPs), nurses and practice managers (PM), (ii) PHN staff who provided direct support to intervention practices, and (iii) provided written informed consent.” Please see page 7, lines 159 – 162 of the revised manuscript.

lines 157-8 - what are these data?

Response – The following sentence has been edited to “After obtaining consent, PEN Computer Systems (PenCS) used their software to extract clinical data on the pre-defined CHD measures (S1 Table) from the intervention practices automatically each month and transmitted them to the study team.” Please see page 8, lines 179 – 182 of the revised manuscript.

lines 177-80 - more detail on these data items would be useful.

Response – The following sentence has been edited to “Details of the data sources are published elsewhere (S1)”. A reference to the publication detailing the data sources has also been added. The published protocol has also been attached as S1 Appendix in the submission. Please see page 9, lines 203 – 204 of the revised manuscript.

line 232 - what level of disagreement was there about themes?

Response – The following sentence has been edited to “Minor Disagreements about the interpretation of some responses and the categorisations of some themes were discussed with a third researcher (KH) until a consensus was reached.” Please see page 12, lines 271 – 273 of the revised manuscript.

PLOS authors have the option to publish the peer review history of their article ([what does this mean?](https://journals.plos.org/plosone/s/editorial-and-peer-review-process#loc-peer-review-history)). If published, this will include your full peer review and any attached files.

If you choose “no”, your identity will remain anonymous but your review may still be made public.

**Do you want your identity to be public for this peer review?** For information about this choice, including consent withdrawal, please see our [Privacy Policy](https://www.plos.org/privacy-policy).

Reviewer #1: No

Reviewer #2: No
